# Standards and guidelines for canine clinical genetic testing laboratories

**DOI:** 10.1007/s00439-018-1954-4

**Published:** 2018-11-13

**Authors:** Lisa G. Shaffer, Kyle Sundin, Anja Geretschlaeger, Julia Segert, June E. Swinburne, Ramon Royal, Robert Loechel, Christina J. Ramirez, Blake C. Ballif

**Affiliations:** 1 Paw Print Genetics, Genetic Veterinary Sciences, Inc., 220 E Rowan, Suite 220, Spokane, WA 99207 USA; 2 Feragen GmbH, Salzburg, Austria; 3 Animal DNA Diagnostics Ltd, Cambridge, UK; 4 VetGen, LLC., Ann Arbor, MI USA

## Abstract

This publication represents a proposed approach to quality standards and guidelines for canine clinical genetic testing laboratories. Currently, there are no guidelines for laboratories performing clinical testing on dogs. Thus, there is no consensus set of protocols that set the minimal standards of quality among these laboratories, potentially causing variable results between laboratories, inconsistencies in reporting, and the inability to share information that could impact testing among organizations. A minimal standard for quality in testing is needed as breeders use the information from genetic testing to make breeding choices and irreversible decisions regarding spay, neuter or euthanasia. Incorrect results can have significant impact on the health of the dogs being tested and on their subsequent progeny. Because of the potentially serious consequences of an incorrect result or incorrect interpretation, results should be reviewed by and reported by individuals who meet a minimum standard of qualifications. Quality guidelines for canine genetic testing laboratories should include not only the analytical phase, but also the preanalytical and postanalytical phases, as this document attempts to address.

## Introduction

More than 150 disease-associated mutations have been identified in the domestic dog and have enabled the use of genetic testing in preconception screening in breeding programs and for making a definitive diagnosis in symptomatic dogs. Although some disease mutations occur in a single breed, many occur in several breeds either indicating a single ancestral founder with the mutation or crossbreeding between breeds with the mutation prior to the designation of the foundation stock. Thus, occurrence and frequencies of specific mutations and their association with disease varies among the different dog breeds depending on when the mutation arose during breed development. Artificial selection for desired traits within certain breeds has inadvertently established deleterious disease-associated mutations in these populations.

Dams and sires used in breeding programs should, at a minimum, be screened for the most common mutations recognized for that breed (Ramirez et al. 2017). Two individuals known to carry the same recessive mutation should not be bred, as to protect against producing homozygous, affected offspring. However, carrier to noncarrier breeding is often encouraged to maintain genetic diversity within a breed. In such breedings, approximately half of the offspring are expected to be heterozygous carriers. Therefore, the offspring should be screened for the mutations carried by the parents and any carriers should be bred only to homozygous wildtype individuals in the future. Such responsible breeding can retain the desired physical and behavioral characteristics for a breed while controlling for the occurrence and spread of inherited diseases within a breed, but this approach is reliant on accurate genetic testing (Ramirez et al. 2017).

Currently, there is no regulatory oversight or testing standards for diagnostic veterinary genetic testing laboratories (Ramirez et al. 2017). In the United States, the American Association of Veterinary Laboratory Diagnosticians provides accreditation for university-based and state-affiliated veterinary pathology laboratories but does not provide minimal guidelines for genetic testing standards. In addition, veterinary genetic testing laboratories are not currently required to be accredited, potentially impacting accurate testing for veterinarians in making diagnoses and can be similarly detrimental to dog owners who may use inaccurate genetic testing information in their breeding programs (Ramirez et al. 2017).

In contrast, for human genetic testing, the American College of Medical Genetics and Genomics (ACMG) has established voluntary standards for clinical laboratories (American College of Medical Genetics and Genomics Standards and guidelines for clinical genetic laboratories 2018), including guidelines on appropriate quality control and quality assurance, regarding sample handling, DNA extraction and testing. In addition, guidelines have been established for proficiency testing and personnel qualifications. For preparation of this document, we have relied on three publications for guidance on minimal standards for genetic testing laboratories (Maddalena et al. 2005; Monaghan et al. 2008, 2013) as well as the ACMG Standards and Guidelines.The following standards and guidelines were developed primarily as a baseline resource for canine clinical genetic testing laboratories to help them provide high-quality genetic testing services. These are minimal standards that should not be considered inclusive of all proper procedures but represent an effort to develop and maintain high technical standards for the performance and interpretation of clinical testing. Each clinical laboratory must apply their own professional judgment when rendering results for any particular patient or specimen. Laboratories must meet minimum standards and strive to achieve desired standards. It is the hope that these baseline standards can be expanded upon to provide more detail to laboratories striving to implement quality assurance and quality control programs.

## General requirements

### Personnel

#### Minimum standard

Each clinical laboratory must have a laboratory director, technical supervisor or medical director on site in the facility who oversees the clinical work, supervises test development and validation, reviews clinical data and signs out the reports. The laboratory director and/or technical supervisor must have an appropriate doctoral degree (PhD, DVM or equivalent) and at least 2 years of training in a clinical genetics laboratory. Medical directors should have their doctorate in veterinary medicine (DVM) and appropriate licenses. Clinical laboratory technologists or technicians must hold an undergraduate degree in a relevant scientific field.

#### Desired standard

The laboratory has both a laboratory director (or technical supervisor) and a medical director on site. The laboratory director and/or technical supervisor has their certification in a relevant field. Examples include, but are not limited to, the American Board of Medical Genetics and Genomics, The Canadian College of Medical Geneticists, the American Board of Pathology or the American Board of Clinical Chemistry. Medical directors and laboratory directors and/or technical supervisors with a DVM have performed their residency and are certified in a subspecialty within veterinary medicine. Examples of board certification bodies include the American Veterinary Medical Association American Board of Veterinary Specialties or the European Board of Veterinary Specialists. Clinical laboratory technologists or technicians, in addition to having an undergraduate degree in a relevant scientific field, should have at least 5 years of relevant laboratory experience.

### Facilities

#### Minimum standard

Laboratory space, equipment and facilities are sufficient to ensure safe, accurate and acceptable standards of performance. All laboratory equipment (including temperature-dependent equipment) is maintained, cleaned and monitored at appropriate intervals. Records of such maintenance are recorded and kept. Standard operating procedures (SOPs) should be in place for sample handling and for minimizing contamination. A significant source of contamination within laboratories can come from amplified products after PCR. Laboratory areas should be designated and physically separated for reagent preparation, sample preparation, and PCR amplification/detection. Separate rooms should be designated for pre-PCR and post-PCR workspaces with appropriate air handling and dedicated equipment. Workflow should ensure unidirectional flow from pre-PCR to post-PCR areas to reduce the possibility of sample contamination.

### Quality practices

#### Minimum standard

A quality manual should be developed and maintained for all laboratory processes and is reviewed annually by all staff and the laboratory director. This manual includes protocols and work instructions for all aspects of the testing process including assay development and validation, specimen handling, receipt and storage, all testing procedures, and data review and reporting. The laboratory has a documented quality management system that includes quality control (QC), quality assurance (QA), quality improvement (QI) and corrective and preventative action (CAPA) plans to assure that all reagents, equipment, methodologies, and personnel operate at optimum levels. These plans must be reviewed annually by the laboratory and/or medical director. In addition, laboratories developing in-house tests must have a development plan, validation plan and SOP for releasing new tests. The development and validation plans may be modified as the test is being developed and validated. The SOP for each test should be reviewed annually and modified if standard practices have deviated from the plan. At a minimum, results of concern or disputed by the customer must be investigated and repeat testing or other actions to confirm the original results when warranted.

#### Desired standard

The results from genetic testing are often used by breeders and veterinarians to make decisions regarding spay, neuter and euthanasia. As such, laboratories should desire the highest accuracy possible. In the absence of an external proficiency program, implementation of a method-based proficiency testing (Schrijver et al. 2014) protocol for each mutation region is the desired standard within a laboratory. This standard requires the use of two independent methods with non-overlapping primers, when possible depending on the genomics region, between the two assays to minimize allele dropout due to unforeseen polymorphisms in individual samples (Ramirez et al. 2017, 2018). If two different methods are not possible, the laboratory must ensure that the two primer sets used in PCR do not contain significant sequence overlap. Results from the two assays are compared and if the genotypes are the same between the two assays, results are reported. If the genotypes are discordant, a third assay is implemented, or a new sample is obtained to repeat the testing. In addition to the minimum standards, the laboratory has a quality committee that regularly meets to review the quality metrics being measured for the laboratory as well as adverse events and non-conforming products, laboratory errors, customer complaints, quality improvement reports, and corrective or preventative actions due to issues identified by the routine quality assurance and quality control practices. The committee also ensures that laboratory staff are properly trained and that the training is documented as part of the overall quality system. Finally, the quality committee performs routine internal audits of the laboratory to ensure that the established quality system is being followed.

### Privacy

#### Minimum standard

Laboratory has a privacy policy that is maintained and reviewed annually. Records are maintained in a manner that ensures privacy for the dog owner. Records are only accessible by designated laboratory staff that have been instructed about the laboratory’s privacy policy. Laboratory records are released only to dog owners/breeders or veterinarians who submitted the samples and/or placed the order on the dog.

#### Desired standard

In addition to the minimum standard, laboratory results are released to third parties only after proper written authorization by the owner, breeder or veterinarian.

## Test validation

### Clinical validation

#### Minimum standard

Critical review of one or more peer-reviewed publications that provide strong evidence that a particular gene mutation causes or is strongly associated with a clinical disease or phenotypic trait. Review should include evaluation of whether there is significant co-segregation of the mutation with the disease or trait, whether the mutation was identified in a single affected individual, single pedigree, found widely within the breed or across many breeds, whether functional studies of the mutation were performed, and whether the test will provide relevant information for diagnosis, prognosis, treatments, breeding decisions, or veterinary surveillance. Data in the publication must be sufficient to determine the sensitivity and specificity of the test being developed. Analytical sensitivity is defined as the proportion of samples with a known mutation that are correctly classified/identified with the at-risk genotype or trait. Analytical specificity is defined as the proportion of samples with no known mutation that are correctly classified/identified with the wildtype (normal) genotype for the disease or trait. Clinical utility, the ability of a clinical test to reliably identify individuals who have or will develop the disorder or trait, should be clear from the publication prior to test development.

#### Desired standard

If the clinical validity is not clear from the publications, the laboratory works with breed clubs and breeders to understand their desire to establish a clinical test for the published mutation and collect samples from normal dogs and from those with the disease or displaying the trait for in-house analytical validation. The clinical specificity of a test should be determined and is defined as the proportion of unaffected individuals who will be identified by the test as negative (normal, homozygous wildtype). When possible, the positive predictive value of a test should be determined, which is the proportion of positive test results that correctly identify individuals with the disease or trait.

### Analytical validation

#### Minimum standard

The analytical validity of a test is defined as its ability to accurately and reliably identify a mutation of interest in the sample type that will be used clinically. Laboratories must validate their in-house assay for each mutation regardless of what is stated in a peer-reviewed publication. The methodology for mutation detection is not critical as long as the laboratory has demonstrated proficiency with that method for other tests being offered. For any specific mutation, the assay must be validated on a number of sample types (cheek swabs, blood, semen, etc.) with known genotypes. Because for some conditions the mutant genotype may be rare and unavailable to assess the analytical sensitivity, the laboratory may offer the new test after validation with normal (wildtype) samples while it continues to collect the test results and correlate those with clinical diagnosis and pedigree history. The limitations of the test must be determined, and any variables identified and monitored for continued high-level performance of the test. Primers and probe sequences should be subjected to BLAST search to identify homologous regions in the genome that might be targets and interfere with the accuracy of the test. Genomic regions of interest should be examined using the public literature and databases for known variation that might interfere with primer binding sites (Ramirez et al. 2018) during primer design. For each test, primers, probes, PCR conditions, expected wildtype and mutation sizes of amplicons, map positions noting autosomal or X-linked, and other specifications regarding the methodologies used for each test must be documented and reviewed on an ongoing basis.

#### Desired standard

In addition to the minimum standard, laboratories should seek to obtain samples from other laboratories, breed clubs and breeders for which the genotypes and/or clinical diagnoses are known for validation of the assay. The laboratory should strive to develop two independent assays for each mutation region as part of their internal method-based proficiency testing program (Schrijver et al. 2014). This allows for any false positive homozygous mutant genotype or any false negative homozygous wildtype genotype to be corrected as well as allows for an alternative testing method should a genotype not correlate with a particular phenotype. The performance of any confirmatory or duplicate testing performed should be tracked and periodically reviewed. Assays should be evaluated for reproducibility as part of the performance standards of each test. Ongoing collection of patient outcomes is encouraged for continual monitoring of the clinical validity of a test.

## Preanalytical standards

Lack of proper preanalytical standards can result in the wrong tests being ordered and potentially cause a missed diagnosis or subsequent breeding resulting in affected puppies.

### Customer education and websites

#### Minimum standard

The laboratory website should include a detailed description of the available disease tests, appropriate breeds, clinical signs of the condition, mode of inheritance, appropriate uses and limitations of the test, turnaround times and pricing. When available, disease variability, prevalence, penetrance, age of onset, treatment options, and life expectancy should be included. For each disease or trait, the gene should be listed and the mutation specified. This will allow for comparisons between laboratories that the same mutation was interrogated, as many genes have more than one mutation that can lead to a specific phenotype. The laboratory director and/or medical director should be available to answer any questions that owners, breeders or veterinarians might have regarding the disease and appropriate uses of the test. Detailed information on acceptable sample types, how to obtain, store and ship the sample, and sample quantity should be listed on the website.

#### Desired standard

In addition to the minimum standards, the laboratory website should display the most commonly used names, abbreviations and acronyms for the disease, condition or trait to try to minimize confusion that may lead to ordering the wrong test. The name may vary between breeds depending on how the breeders of a specific breed refer to that disease or trait. Laboratories should work together for uniformity for disease names, abbreviations and acronyms across all testing laboratories. The laboratory website should include any differential diagnoses that could be considered, the clinical sensitivity, and positive predictive values for each test when available. For certain diseases, no causative mutation has been identified, but there may be tests available for linked markers or risk alleles. Such tests should be clearly identified and labeled as associated markers on the website.

### Pretest counseling

#### Minimum standard

The laboratory should have qualified individuals available to assist breeders, owners and veterinarians with ordering the appropriate tests. These individuals should have detailed knowledge about which mutations are present in any given breed. This knowledge is particularly pertinent for diseases or traits in which there may be several breed specific alleles, as well as cases in which there may be more than one causative allele within a given breed. Questions about clinical validity and sensitivity should be referred to the laboratory director and/or medical director or other appropriately trained personnel such as genetic counselors.

### Specimens

#### Minimum standard

Written laboratory standards for acceptance or rejection of specimens must be in place including optimal and acceptable specimen types, variables that affect acceptability including insufficient quantity, exposure to extreme temperatures and inappropriate collection modes. Samples should arrive in the laboratory labeled with two identifiers, which may include the dog’s name, microchip number, permanent tattoo, date of birth, laboratory number or order number. In addition, all samples should include the breed (if known), sex, and pertinent family history including related dogs that have been tested by that laboratory. For samples lacking sufficient information or unique identifiers, the laboratory should contact the ordering owner, breeder or veterinarian for the missing information. Accompanying the samples should be a requisition form that indicates the tests desired and identifies the ordering individual with contact information in case there are questions.

#### Desired standard

Orders are reviewed for appropriateness of the requested tests based on the breed being tested. Ordering individual is contacted when the sample is received by the laboratory. Date and time are stamped on the requisition form and recorded in the laboratory management system (LMS) as received. Missing information or unique identifiers are recorded and noted on the final laboratory report.

## Analytical standards

### Controls

#### Minimum standard

A no template control should be included in each assay to detect contamination. Negative controls (homozygous wildtype) should be included each time an assay is performed. It may not be possible to include a positive control for each mutation but representative positive controls for each method should be run periodically as needed for quality assurance. Every effort should be made to include positive controls as they become identified in the laboratory.

#### Desired standard

Residual clinical samples demonstrating the mutation should be retained as positive controls for ongoing quality assurance and future test development.

### Sample preparation

#### Minimum standard

Specimens are handled and processed one at a time to prevent contamination, tampering or sample mix-up. Judgement about sample quality is made at the time of sample receipt by the laboratory. Sample type (blood, cheek swab, tissue, semen, etc.) is recorded in the LMS noting amount and gross quality of the sample. Submitting owner, breeder or veterinarian is contacted if the specimen does not meet laboratory requirements or if the sample appears compromised in any way (i.e., visible mold, mildew, bacterial growth, dirt, food particles for cheek swabs, clotted or lysed blood for blood samples). A laboratory SOP is in place for handling unacceptable specimens. The laboratory has a sample retention policy regarding the original sample and extracted DNA and retains any remaining original sample at least until all testing is completed and the report has been signed out.

#### Desired standard

In addition to the minimum standard, the laboratory retains any remaining original sample for 6 months and the extracted DNA for 3 years.

### Validation of methods

#### Minimum standard

Regardless of whether the assay is developed using an in-house method or purchased as a kit, the laboratory must perform validation to demonstrate that the assay is detecting the appropriate mutation and wildtype sequence. Even if a method is being used in the laboratory for other tests, the assay developed and specifically designed to detect a certain mutation associated with a disease or trait must be validated and its analytical specificity and analytical sensitivity must be determined. Multiplex assays must be validated, demonstrating that all mutation regions are successfully amplified and expected results are obtained before the test can be offered.

## Postanalytical standards

### Reporting

#### Minimum standard

The following demographics should be reflected on the reports: order date, receipt date of the sample by the laboratory, date reported, the name of the person who ordered the test and/or the owner, breeder or veterinarian’s name, unique laboratory number of the sample and order number, dog’s call name, breed (if known), sex and date of birth. Reports should include the disease name, gene tested, specific mutation interrogated, results obtained after genotyping for each gene region tested, a statement interpreting the data, and any recommendations or follow-up testing required. Reports should be signed by the laboratory director, technical director and/or medical director. Test limitations should be stated. Reports should be issued on letterhead with the contact information for the laboratory.

#### Desired standard

In addition to the minimum standard, reports should include notes regarding any verbal results to the customer, amended reports, corrected reports or notes regarding any deviation from the laboratory’s established standard practices. Tests that were sent to another laboratory for testing should be noted on the laboratory report with the send-out laboratory’s contact information. Unpublished, nonpeer-reviewed mutations must be identified on the report as investigational. Both the laboratory director/technical director and medical director should review results and sign out reports. Laboratories should work together to define acceptable nomenclature for the genotype results on reports, as these are not currently standardized across the industry.

### Interpretations and disclaimers

#### Minimum standards

The laboratory website and the report should state that normal results do not exclude any undetected mutation that may be present in the gene that was tested or any other gene that was not tested. In addition, even in the presence of an internal method-based proficiency testing (Schrijver et al. 2014), the laboratory website and report should make clear that the presence of unexpected variation at a primer binding site could lead to allele dropout or preferential amplification of the other allele (Turba et al. 2017). Primers that yield such results are to be removed from use and testing protocols. The disclaimer should include that the presence of mosaicism may not be detected with certain assays and that non-paternity may lead to unexpected results.

#### Desired standards

In addition to the minimum standard, the laboratory’s clinical sensitivity and clinical specificity for each assay should be shown on the laboratory website and on the laboratory report.

### Retention of records and security

#### Minimum standard

The laboratory has a record retention policy. All records are maintained in a manner that will ensure privacy, security, integrity and access. The laboratory computer system has been validated for security and performance including appropriate hardware, software, and back-up systems to allow uninterrupted functioning of the laboratory and prevention of data loss or security breaches.

#### Desired standard

In addition to the minimum standard, record retention and access is maintained for a period of at least 20 years.

## Summary

These standards and guidelines form the basis for detailed procedures within each individual laboratory. The laboratory is responsible for critically evaluating the peer-reviewed literature and determining the clinical validity of tests to develop and offer. The laboratory is responsible for the analytical validation of all in-house testing being offered and should request the validation documentation for any tests that they send to other laboratories to perform. Test results require expert interpretation by an appropriately trained individual. The owner, breeder or veterinarian should work with their laboratory to ensure high-quality testing through discussions about clinical utility of certain tests and bringing any discrepancies or results of concern to the laboratory’s attention. It is the duty of the laboratory to take such concerns seriously and to work with the customer to resolve any issues, including retesting the dog or performing paternity testing to resolve conflicts. Ultimately, laboratories should work together to improve and enhance these guidelines, share information to transition tests from research to clinical testing, and be involved in the ongoing assessment of clinical validity and clinical utility of certain tests being offered. Next steps should be the design and implementation of a voluntary external proficiency program designed for quality improvement across all canine genetic testing laboratories.
